# Clinical application of directional dilation in transurethral columnar balloon dilation of the prostate

**DOI:** 10.12669/pjms.38.6.5235

**Published:** 2022

**Authors:** Ming Zhang, Jianghua Jia, Qingsong Meng, Dongbin Wang

**Affiliations:** 1 Ming Zhang, Department of Urology, The Second Hospital of Hebei Medical University, NO. 215 Heping Xi road, Shijiazhuang, Hebei, 050000, China; 2 Jianghua Jia, Department of Urology, The Second Hospital of Hebei Medical University, NO. 215 Heping Xi road, Shijiazhuang, Hebei, 050000, China; 3 Qingsong Meng, Department of Urology, The Second Hospital of Hebei Medical University, NO. 215 Heping Xi road, Shijiazhuang, Hebei, 050000, China; 4 Dongbin Wang, Department of Urology, The Second Hospital of Hebei Medical University, NO. 215 Heping Xi road, Shijiazhuang, Hebei, 050000, China

**Keywords:** Directional dilation, Prostatic hyperplasia, Columnar balloon dilation

## Abstract

**Objectives:**

To investigate the clinical efficacy and safety of directional dilation in transurethral columnar balloon dilation of the prostate (TUCBDP), and summarize relevant experience.

**Methods:**

Retrospective analysis was performed on the clinical data of 38 patients with prostatic hyperplasia admitted to the Department of Urology, The Second Hospital of Hebei Medical University from October 2017 to January 2020 who underwent TUCBDP with directional dilation (12 o’clock direction). Complications related to surgery including hemorrhage, urinary incontinence and pain were analyzed. Moreover, patients were followed up for six months postoperatively, and their preoperative and postoperative maximum urine flow rate (Qmax), residual bladder volume (PVR), international prostate symptom score (IPSS), and quality of life score (QoL) were compared.

**Results:**

Thirty-eight patients underwent TUCBDP successfully, with dilation positions all in the 12 o’clock direction and no ectopic dilation point. All patients had no severe hematuria postoperatively. Numeric rating scales (NRS) was utilized twice at 8h and 24hour postoperatively to score the pain degree, with no statistically significant difference (P=0.157). Hb was reexamined on the first day postoperatively, with no statistically significant difference compared with that preoperatively (P>0.05). The bladder irrigation time was 1-2 Day postoperatively, while the urethra was removed five days postoperatively, with no severe hematuria in all patients. Two patients developed mild urinary incontinence, which disappeared on the 2nd and 5th day after extubation, respectively, while no patients had dysuria and urinary retention. All 38 patients were detected for Qmax and PVR after urethral removal, with a statistically significant difference compared with those preoperatively (P<0.001), and were reexamined three months postoperatively for Qmax and PVR, with a statistically significant difference compared with those postoperatively (P<0.001); IPSS and QoL were significantly different from those preoperatively with statistically significance (P<0.001). At the follow-up six months postoperatively, Qmax, PVR and IPSS showed statistically significant differences compared with that at three months postoperatively (P<0.05), while QoL showed no statistically significant differences compared with that at 3 months postoperatively (P=0.088).

**Conclusion:**

Directional dilation is improved in TUCBDP as having the advantages of safety and effectiveness, and it is worthy of clinical promotion.

## INTRODUCTION

Benign prostate hyperplasia (BPH), as one of the most common diseases in urology, usually makes inroads on middle-aged and elderly men over the age of 50. With the advent of an aging society, the incidence of BPH is increasing year by year, seriously affecting the quality of life of patients. For a long time, transurethral resection of prostate (TURP) has been considered as the gold standard for the treatment of BPH.^1^ However, some patients with BPH are relatively young and still have normal sex life needs, while other patients are unable to tolerate long-term surgery due to their age and multiple underlying diseases such as heart and lung; TUCBDP is considered as a better treatment option for these patients due to its simplicity of operation, short duration of surgery, and minimal impact on sexual function.

TUCBDP achieves the effect of surgical treatment by dilating the weakest part of the prostatic capsule (mostly in the 12 o’clock direction). In view of the large individual differences in patients with BPH, different positions of dilation points may occur during surgery, which will affect the surgical effect to some extent and may cause injuries to other parts of the urethra. In this study, the feasibility of TUCBDP was investigated by cutting the 12 o’clock position of the prostate in advance to artificially weaken the part, so that the 12 o’clock position can be dilated as planned during surgery without damage to other positions, as well as evaluating the surgical outcome.

## METHODS

A total of 38 patients were enrolled in this study, ranging from 54 to 89 years old, with an average of 73.2±8.85 years old. Preoperative detection of Hb was 89-135g/L, with an average of 111.53±3.61g/L; Prostate volume was 35.13-95ml, with an average of 68.26±14.99ml; Qmax was 3-7ml/s, with an average of 4.68±1.09ml/s; PVR was 45-162ml, with an average of 79.45±26.87ml, IPSS was 19-31 points, with an average of 25.11±1.03 points; QoL was 4-6 points, with an average of 5.37±0.23 points. All patients were confirmed to have bladder outlet obstruction (BOO) by urodynamic examination preoperatively. And all patients were diagnosed as middle prostatic protrusion into the bladder by color ultrasound before surgery.

### Ethical Approval

The study was approved by the Institutional Ethics Committee of The Second Hospital of Hebei Medical University on June 20, 2017 (No.2017-R101), and written informed consent was obtained from all participants.

### Exclusion Criteria

Neurogenic bladder, severe urethral stricture, severe coagulation dysfunction, complicated bladder calculi, suspected prostate cancer, etc.

### Surgical Methods

**Fig.1:**
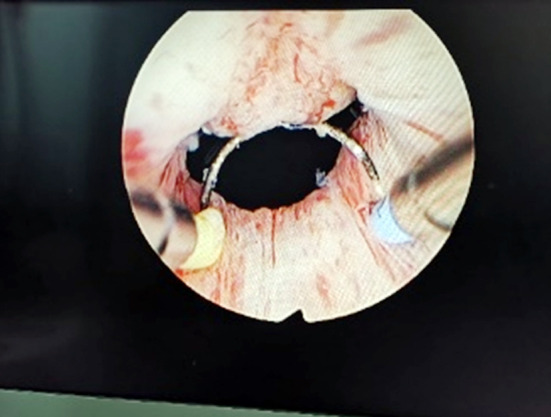
The prostate was dissected longitudinally at 12 o ’clock position.

**Fig.2:**
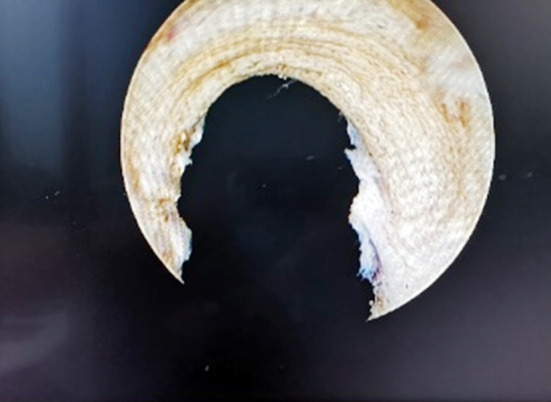
Annular fibers were fully exposed.

**Fig.3:**
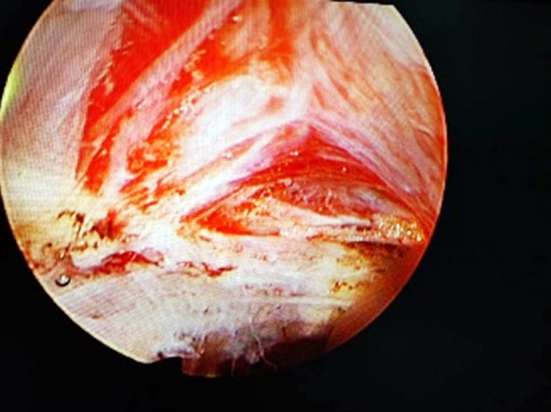
Satisfactory prostatic dilation was observed at 12 o ’clock position.

All patients were subjected to intraspinal block anesthesia, lithotomy position, electroresection endoscopic placement, and routine observation of the conditions in the prostatic urethra and bladder cavity, so as to further exclude tumors, stones and other lesions. Plasma electrosurgical loops were placed on all patients, and their prostate tissues were cut longitudinally at the 12 o’clock position of the prostate to expose the circular fibers (Fig1 & Fig.2). Adequate hemostasis was then performed and the electroscope was withdrawn after bladder filling. A suitable prostate dilation catheter was selected and inserted from the urethra according to the volume of the prostate. The dilation catheter was placed in a suitable position, the dilation operation was performed routinely, and the urination test was performed after the catheter was withdrawn, all of which were positive. Finally, electroscopy was re-placed and a satisfactory dilation of the prostate at the 12 o’clock position was observed with visible fat (Fig.3). Adequate electrocoagulation was performed. For patients with protruding middle lobe, the middle lobe was flattened, the scope was withdrawn, the F20-22 three-lumen catheter was indwelled, and bladder irrigation was continued.

### Postoperative observation and follow-up

Bladder irrigation was continued for 1-2d postoperatively, and the irrigation speed was adjusted at any time according to the color of the irrigation. The degree of pain was assessed by NRS at eight hours after anesthesia and was reassessed at 24hour after anesthesia. The routine examination was performed on the first day postoperatively to understand Hb situation compared with that preoperatively. The urinary catheter was removed five days postoperatively to observe the presence of severe hematuria, urinary incontinence or urinary retention, and Qmax and PVR were detected. Urinary incontinence is defined as the uncontrolled outflow of urine from the bladder.^2^ Qmax, PVR, IPSS and QoL were reexamined three and six months postoperatively. Examination would be initiated when subjects feel they have reached their maximum willingness to urinate and urinate into a urine collector. The physician obtained the maximum urine flow rate from the urination/volume fit diagram, which was defined as Qmax. B ultrasonography was performed immediately after urination to determine residual urine volume in the bladder, which was defined as PVR.

### Statistical Analysis

All data in this study were processed by SPSS 21.0 statistical software, measurement data were expressed as mean ± standard deviation (), and a paired t test was performed between the two groups. P<0.05 indicates a statistically significant difference.

## RESULTS

All the 38 patients enrolled successfully completed surgery according to the established plan, and all the dilation point were in the direction of 12 o’clock. None of the patients had dilation or injury in other positions, or had a change of procedure or unplanned reoperation. The surgery time was 12-26 minutes, with an average of 17.46±3.02 minutes, and the estimated intraoperative blood loss was 5-20ml, with an average of 10.35±3.19ml. The NRS score was 0.29±0.45 at 8h postoperatively and 0.21±0.41 at 24h postoperatively, with no statistically significant difference between the two NRS scores (Table-I). Blood routine examination was performed on the first day postoperatively to show that Hb was 105.13±11.67g/L, which had no statistical significance compared with 111.53±11.16g /L preoperatively (P> 0.05) (Table-II). The bladder irrigation time was 1-2Day postoperatively, while the urethra was removed five days postoperatively, with no severe hematuria in all patients. Two patients developed mild urinary incontinence, which disappeared on the 2nd and 5th day after extubation, respectively, while no patients had dysuria and urinary retention. Qmax of 18.76±3.86ml/s and PVR of 23.58±11.20ml were respectively detected in all patients after catheter removal, with statistically significant differences compared with those preoperatively (Table-III). Thirty eight patients showed Qmax of 22.50±3.08ml/s, PVR of 9.66±2.53ml, IPSS of 6.03±1.48 points and QoL of 0.74±0.75 points at the 3-month follow-up. Qmax and PVR were significantly different from those postoperatively with statistically significance (Table-IV), while IPSS and QoL were significantly different from those preoperatively with statistically significance (Table-V). At the follow-up six months postoperatively, Qmax, PVR and IPSS were 23.61±2.90 mL/s, 5.37±2.79ml and 4.58±1.62, respectively, showing statistically significant differences compared with those at three months postoperatively (P<0.05), while QoL showed no statistically significant differences compared with that at 3 months postoperatively (Table-VI). Five of the patients resumed sexual life 3-5 months postoperatively, with no symptoms of retrograde ejaculation or erectile dysfunction.

**Table I:** Comparison of pain scores at 8h and 24h after surgery [(*x̅*±S), points].

Group	Pain score
8h after surgery (n=38)	0.29±0.45
24h after surgery (n=38)	0.21±0.41
t	1.00
P	0.324

**Table II:** Comparison of Hb before and after surgery [(*x̅*±S), g/L].

Group	Hb
Before surgery (n=38)	111.53±11.16
After surgery (n=38)	105.13±11.67
t	1.4871
P	0.0867

**Table III:** Comparison of PVR and Qmax before and after surgery (*x̅*±S).

Group	PVR (ml)	Qmax (ml/s)
Before surgery (n=38)	79.45±26.87	4.68±1.09
After surgery (n=38)	23.58±11.20	18.76±3.86
t	15.269	-25.235
P	<0.001	<0.001

**Table IV:** Comparison of PVR and Qmax after surgery and 3 months after surgery (*x̅*±S).

Group	PVR (ml)	Qmax (ml/s)
After surgery (n=38)	23.58±11.20	18.76±3.86
3m after surgery (n=38)	9.66±2.53	22.50±3.08
t	7.879	-5.36
P	<0.001	<0.001

**Table V:** Comparison of IPSS and QoL before and 3 months after surgery (*x̅*±S).

Group	IPSS (points)	QoL (points)
Before surgery (n=38)	25.11±1.03	5.37±0.23
3m after surgery (n=38)	6.03±1.48	0.74±0.75
t	34.71	27.18
P	<0.001	<0.001

**Table VI:** Comparison of PVR, Qmax, IPSS, QoL at 3m and 6m after surgery (*x̅*±S).

Group	PVR (ml)	Qmax (ml/s)	IPSS (points)	QoL (points)
3m after surgery (n=38)	9.66±2.53	22.50±3.08	6.03±1.48	0.74±0.75
6m after surgery (n=38)	5.37±2.79	23.61±2.90	4.58±1.62	0.53±0.64
t	7.405	-2.470	3.309	1.539
P	<0.001	0.018	0.002	0.132

## DISCUSSION

BPP is the main cause of urethral outlet obstruction and lower urinary tract stimulation in middle-aged and elderly male patients.^3^ It not only affects the quality of life of patients, but also causes damage such as upper urinary tract hydrocephalus in severe cases, and even affects the patients’ bilateral renal function.^4^ TURP is regarded as a classic surgical method for the treatment of BPP, but it is accompanied by various surgical risks, such as large blood loss, lengthy surgery time and increased perioperative complications for elderly patients with multiple underlying diseases such as heart and lungs.^5^ For this reason, in this study, the operation scheme was further optimized on the basis of the traditional dilation. TUCBDP was used in this study. A plasma electrosurgical loop was first placed to remove part of the prostate tissue in the 12 o’clock direction to further make this part the weakest of the prostate capsule. In this way, the dilation effect is ensured, and the situation that the surgical effect cannot be guaranteed due to the failure of dilation or the occurrence of dilation in other positions is prevented. All the 38 patients enrolled underwent incision of prostate tissue at the 12 o’clock position before conventional dilation to weaken the prostate capsule, and then underwent conventional dilation.

It has been found in related studies^6^ that for high-risk elderly patients with both severe cardiovascular and cerebrovascular diseases and lung diseases, the length of anesthesia and surgery time has a clear bearing on the probability of postoperative complications. With shorter duration of surgery and anesthesia, the risk of perioperative surgery can be effectively reduced and the function of urination can be restored as soon as possible. In this study, the operation time of all patients was 12-26 minutes, with an average of 17.46±3.02 min; Intraoperative blood loss was estimated at 5-20ml, with an average of 10.35±3.19ml. And no patients had dysuria and urinary retention. These results indicate that TUCBDP has the advantages of short operation time and less intraoperative bleeding, and can effectively reduce the risk of perioperative surgery, which is consistent with the results of Wang et al.^7^ It was reported in a study conducted by Cornu et al.^5^ that TURP has a high retrograde ejaculation rate of about 65-70%, while in a study by Poulakis et al.^8^, the incidence of erectile dysfunction after TURP was reported to be about 12%. Burhene et al.^9^ first reported animal experiments and clinical studies on the treatment of BPH by balloon dilation of the prostate as early as 1984, and in 1987, Castaneda et al.^10^ applied prostatic dilation catheter to treat patients with BPH. In this study, five patients with sexual needs resumed sexual life 3-5 months after surgery without retrograde ejaculation or erectile dysfunction. The reason for this difference may be that traditional electroresection surgery may damage the blood vessels and pelvic nerve plexus that innervate the erection of the penis owing to factors such as perforation of the prostate capsule and thermal injury,^11^ supplemented by the influence on the sexual nerves during the treatment of the tip of the prostate, resulting in postoperative sexual dysfunction. While TUCBDP has the advantages of simple operation, rapid operation, minimal trauma and quick recovery. And the operation does not damage the bladder neck and glands, avoiding the occurrence of ejaculation and erectile dysfunction. It was also found in some subsequent studies^12^-^14^ that for elderly BPH patients with underlying diseases such as cardiovascular and cerebrovascular diseases, balloon dilation of the prostate has been put into practice at home and abroad for its advantages of simple operation and rapid operation.

TUCBDP has gradually become more widely used in clinical practice. In the postoperative follow-up of this study, the differences of PVR, Qmax and IPSS at six months, three months and extubation were statistically significant, indicating that the operation achieved satisfactory surgical results, and the recovery effect was better with the extension of time. It’s probably because that TUCBDP dilates the weakest part of the prostate capsule (usually in the 12 o’clock direction) to avoid unnecessary blood vessels, pelvic plexus and sexual nerve damage to the largest extent, so that both lobes of the prostate are displaced laterally and downward, and the fat, fascia tissue and some blood clots of the posterior pubis fill the tissue space, resulting in the failure of the closure of the prostate gland and capsule, so as to ensure the unobstructed urethra. At the same time, damage to the bladder neck can be avoided to ensure its integrity. In the process of urination, the pressure in the urethra is reduced due to its dispersion in all directions, thereby reducing the resistance to urination and ensuring smooth urination.^15^ It was also reported in the study by Huang et al^16^ that TUCBDP has advantages in reducing lower urinary tract syndrome and urethral remodeling. Studies have found that most of the reports in the literature of TUCBDP are conventional dilation operations directly after catheter placement. It was found in the early stage of this operation in our hospital that the dilation point of some patients was not in the 12 o’clock direction, but the posterior lobe of the prostate or combined with bladder neck injury, which may not guarantee the surgical effect, and some patients still need to change the surgical method.

QoL showed no statistically significant differences compared with that at three months and six months postoperatively, indicating that the quality of life of all enrolled patients had recovered at three months postoperatively. The above conclusions are consistent with the report in the literature that TUCBDP can significantly improve IPSS, QoL score, Qmax and PVR^17^, and is superior to traditional TURP in terms of indications, surgery time, operative blood loss and postoperative complications.^18^ It is known^19^ that patients with obvious prostatic middle lobe protrusion also have more severe obstruction symptoms. In order to ensure a satisfactory surgical outcome, all the enrolled patients with middle lobe protrusion underwent middle lobe trimming.

### Limitations of this study

Too few patients with regular life were enrolled hence it was impossible to accurately evaluate the efficacy of TUCBDP, so a larger sample size should be included in the later stage to further support the above conclusions.

## CONCLUSION

To put it in a nutshell, directional dilation is boasted in TUCBDP as having the advantages of accurate clinical efficacy and satisfactory surgical outcomes, which is worthy of clinical promotion.

### Authors’ Contributions:

**MZ,** **DW:** Designed and performed the experiments, wrote the manuscript, and are responsible and accountable for the accuracy and integrity of the work.

**QM,** **JJ:** Conducted the experiments and revised the manuscript.

All authors have read and approved the final manuscript.
